# Correlation between the metabolic profile of Nelumbo Seed, a component of Seishinrenshiin, and its inhibitory activity on bladder smooth muscle contraction

**DOI:** 10.1007/s11418-025-01889-4

**Published:** 2025-03-19

**Authors:** Kazuo Harada, Yuki Fukuda, Takahiro Ohkubo, Kimio Sugaya, Yukihiko Osaki

**Affiliations:** 1 Graduate School of Pharmaceutical Sciences, Osaka University, Yamadaoka 1-6, Suita, Osaka 565-0871 Japan; 2 Central R&D Laboratory, KOBAYASHI Pharmaceutical Co., Ltd, 1-30-3 Toyokawa, Ibaraki, Osaka 567-0057 Japan; 3 Southern Knights’ Laboratory Co., Ltd, 1-1-823 Miyagi, Chatan, Okinawa 904-0113 Japan

**Keywords:** Nelumbo Seed, Neferine, Bladder smooth muscle, Metabolic profiling, Seishinrenshiin

## Abstract

Seishinrenshiin (SRI), a Kampo formula, is often used for frequent urination. Nelumbo Seed, a crude-drug component of SRI was reported to inhibit bladder smooth muscle contraction using excised rat bladder tissues. However, the active ingredients of Nelumbo Seed have not yet been identified. In this study, we investigated the active ingredients of Nelumbo Seed that inhibit bladder smooth muscle contraction. We obtained liquid chromatography/mass spectrometry profiles of extracts prepared from five types of Nelumbo Seed materials from different production areas and evaluated their inhibitory effects on excised rat bladder smooth muscle contraction. Analysis of these data using orthogonal projections to latent structures revealed neferine (Nef) as the compound with the highest variable influence on projection. Among the Nelumbo Seed materials processed using different methods, the Nef content was the highest in ‘Sekirenshi’, when embryo and pericarp were not removed at all. We determined the effects of administering Nelumbo Seed extracts with different Nef content on frequent-urination model rats. The Nef content in rat plasma was the highest when the Sekirenshi extract was administered, but Nef was not detected when seed embryos were removed. In this model, administration of the Nelumbo Seed extract improved the maximum bladder contraction pressure and bladder contraction interval, albeit not significantly. In particular, Sekirenshi tended to reduce the maximum bladder contraction pressure compared to other Nelumbo Seed. Our results indicate that although Nef in Nelumbo Seed does not clearly improve frequent urination, it might contribute to the improvement of urination disorder.

**Graphical Abstract:**

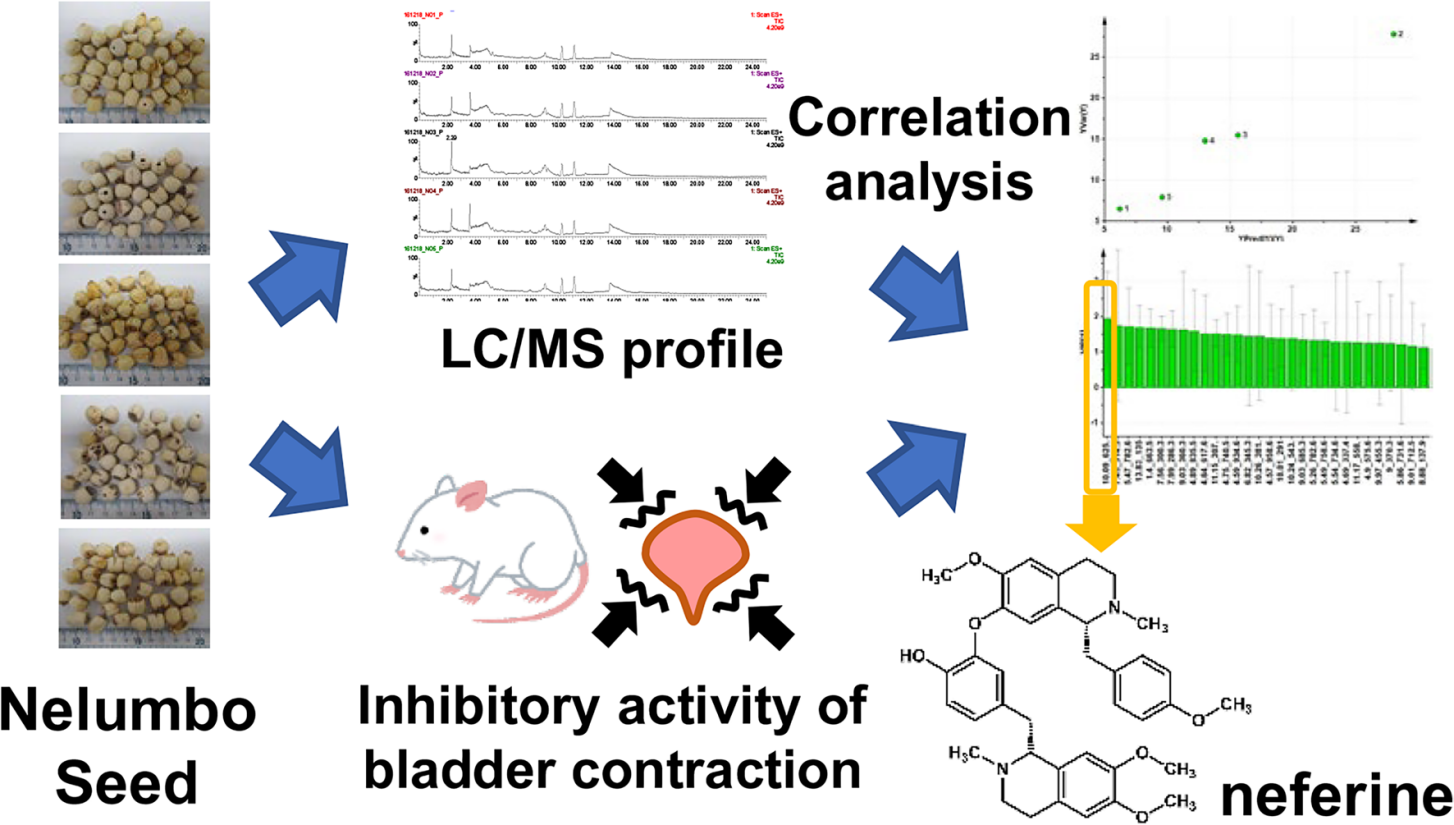

**Supplementary Information:**

The online version contains supplementary material available at 10.1007/s11418-025-01889-4.

## Introduction

Urinary disorders caused by an overactive bladder, such as nocturia, urinary urgency, and urge urinary incontinence, are more likely to occur with age and reduce the quality of life [1–4]. The incidence of urinary disorders is expected to increase with the increase in the elderly population in developed countries. Anticholinergic drugs, such as solifenacin, which inhibit bladder smooth muscle contraction, are commonly used for the treatment of urinary disorders [5–8]. In Kampo medicines, ‘Choreito’, ‘Goshajinkigan’, ‘Hachimijiogan’, ‘Ryutanshakanto’, ‘Gorinsan’ and ‘Seishinrenshiin (SRI)’, are often used for treating these disorders [9–11]. SRI (Qing Xin Lian Zi Yin) was included in ‘Taiping Huimin He ji jufang’, which was compiled by the Song Dynasty in ancient China and has been reported to be effective against urinary problems caused by irritability or mental fatigue [10]. Even in modern clinical practice, SRI has been confirmed to be effective in treating urinary disorders. The administration of SRI for 2 to 12 weeks to 18 patients with unidentified complaint due to lower urinary tract disorders resulted in an efficacy rate of 81% in cases showing a ‘deficiency pattern’ [12]. In another study, SRI administration to 35 male patients of chronic prostatitis and urethritis with various equivocal complaints of the lower urinary tract and to 7 female patients exhibiting cystitis symptoms but normal urination, resulting in a satisfactory efficacy rate of 41% in patients with constitutionally infirm stomach and intestines [13]. Among 24 patients with refractory chronic aseptic prostatitis and prostatic pain, 68.8% and 87.5%, respectively, were treated effectively based on the attending physician’s diagnostic index, upon SRI administration for 28 days [14].

SRI is extracted from a mixture of the following crude drugs: Nelumbo Seed, Ophiopogon Root (or Tuber), Poria Sclerotium, Ginseng Root, Plantago Seed, Scutellaria Root, Astragalus Root, Lycium Bark, Glycyrrhiza Root [15]. SRI suppressed the contraction of excised rat bladder smooth muscles. Furthermore, the inhibitory effect on bladder smooth muscle contraction was significantly attenuated when SRI extracts were prepared by removing one of the crude drugs, Scutellaria Root, Nelumbo Seed, Astragalus Root, Lycium Bark, Glycyrrhiza Root, or Plantago Seed [16]. Among these, baicalin and baicalein present in Scutellaria Root were reported to have smooth muscle-relaxing effects [17]. However, the active ingredient of Nelumbo Seed, the second most prominent component of SRI after Scutellaria Root, has not been identified.

Because crude drugs are natural products, their chemical composition and biological activity are greatly influenced by the region of production, harvest time, and post-harvest processing. The active ingredients that directly affect the quality of natural products including crude drugs can be identified by collecting the different materials and comparing their compositions and biological activities, as has been demonstrated in several studies [18–22].

In this study, we collected Nelumbo Seed materials from different regions and compared their liquid chromatography/mass spectrometry (LC/MS) profiles employing an ex vivo test using excised rat bladder specimens to identify the active ingredients that inhibit bladder smooth muscle contraction. Furthermore, we identified the factors that increase the content of the active ingredient and investigated the effects on the blood concentration and bladder in rats administered Nelumbo Seed extract with high content of the active ingredient.

## Materials and methods

### Materials

**Fig. 1:**
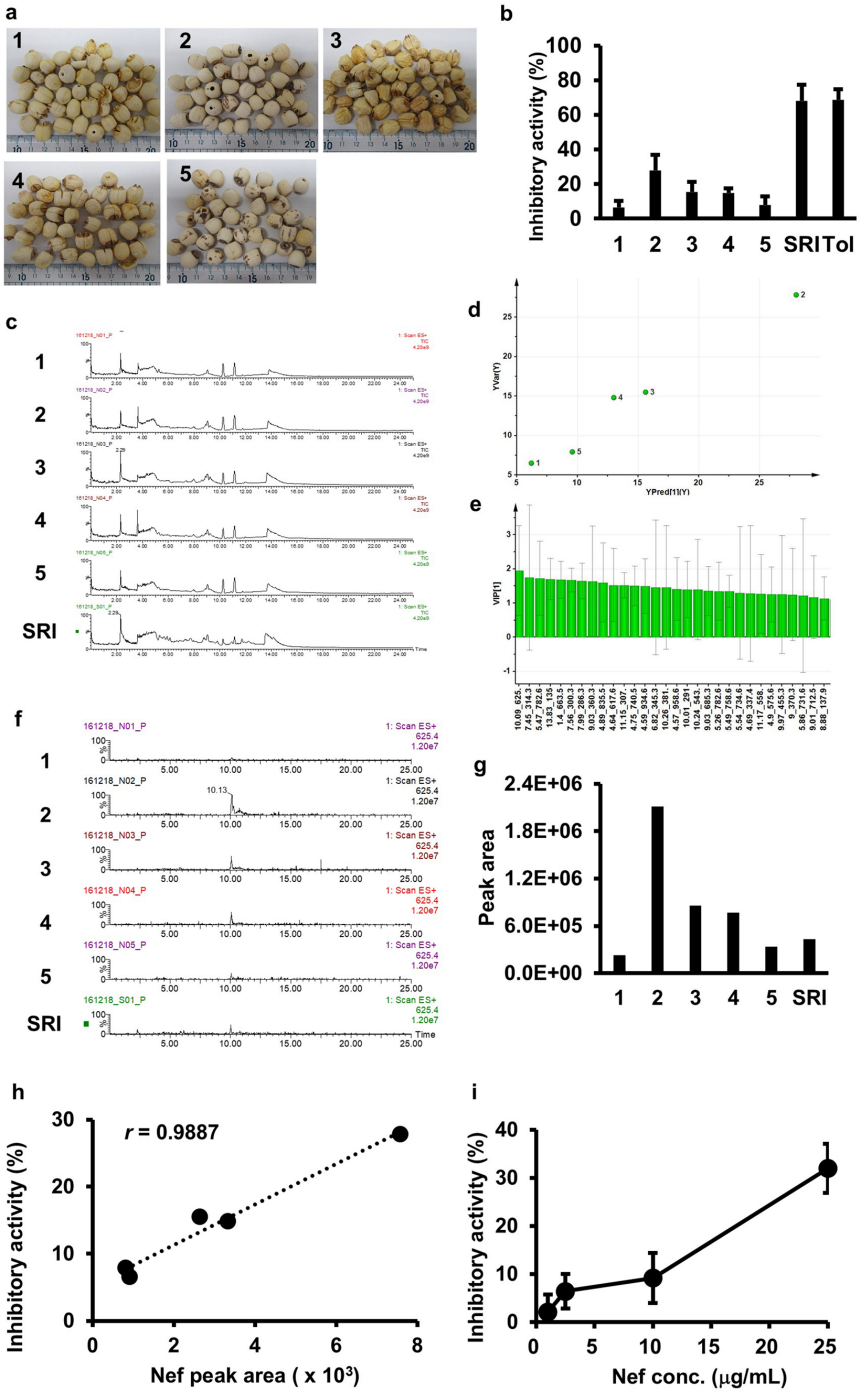
Inhibitory activity assay of bladder smooth muscle contraction and LC/MS metabolic profiling of Nelumbo Seed extract. **a** Photographs of the samples used in this study. The numbers are the same as those in Table 1. **b** Assay of the inhibitory activity of the extract on rat-excised bladder smooth muscle contraction. 0 and 100% of inhibitory activity indicate that the contraction length when the excised bladder was treated simultaneously with the Nelumbo Seed extract and carbamylcholine was the same as when treated with carbamylcholine alone, and was not observed, respectively. SRI: Seishinrenshiin; Tol: tolterodine (100 ng/mL, positive control). **c** LC/MS total ion current chromatograms of Nelumbo Seed extracts. **d**, **e** Results of OPLS between LC/MS peaks and rat-excised bladder smooth muscle contraction inhibitory activity. **d** Y fit, **e** VIP. **f** Mass chromatograms of *m/z* 625 from Nelumbo Seed extracts. **g** Area of *m/z* 625 peak at 10.1 min, assigned to neferine. The value on the vertical axis is an arbitrary value. **h** Scatter plot between neferine peak area and rat-excised bladder smooth muscle contraction inhibitory activity of Nelumbo Seed extracts. **i** Rat-excised bladder smooth muscle contraction inhibitory activity of neferine standard

**Fig. 2:**
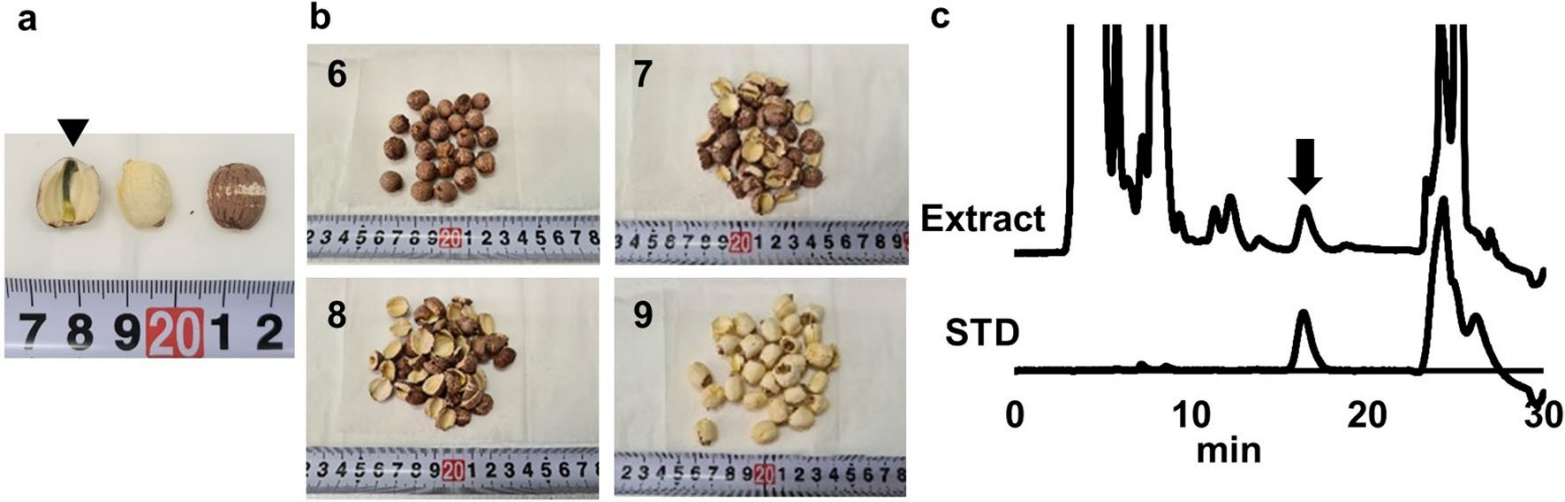
Localization of neferine accumulation in Nelumbo Seed. **a** Photograph of Nelumbo Seed. Left, slice of seed; arrow-head shows the embryo. Middle, outside of the seed slice without the endocarp; Right, Nelumbo Seed without any treatment. **b** Photographs of the materials used in study on neferine accumulation in Nelumbo Seeds. **c** HPLC chromatograms of Nelumbo Seed extract (upper) and neferine standard solution (lower). Arrow indicates neferine

**Fig. 3:**
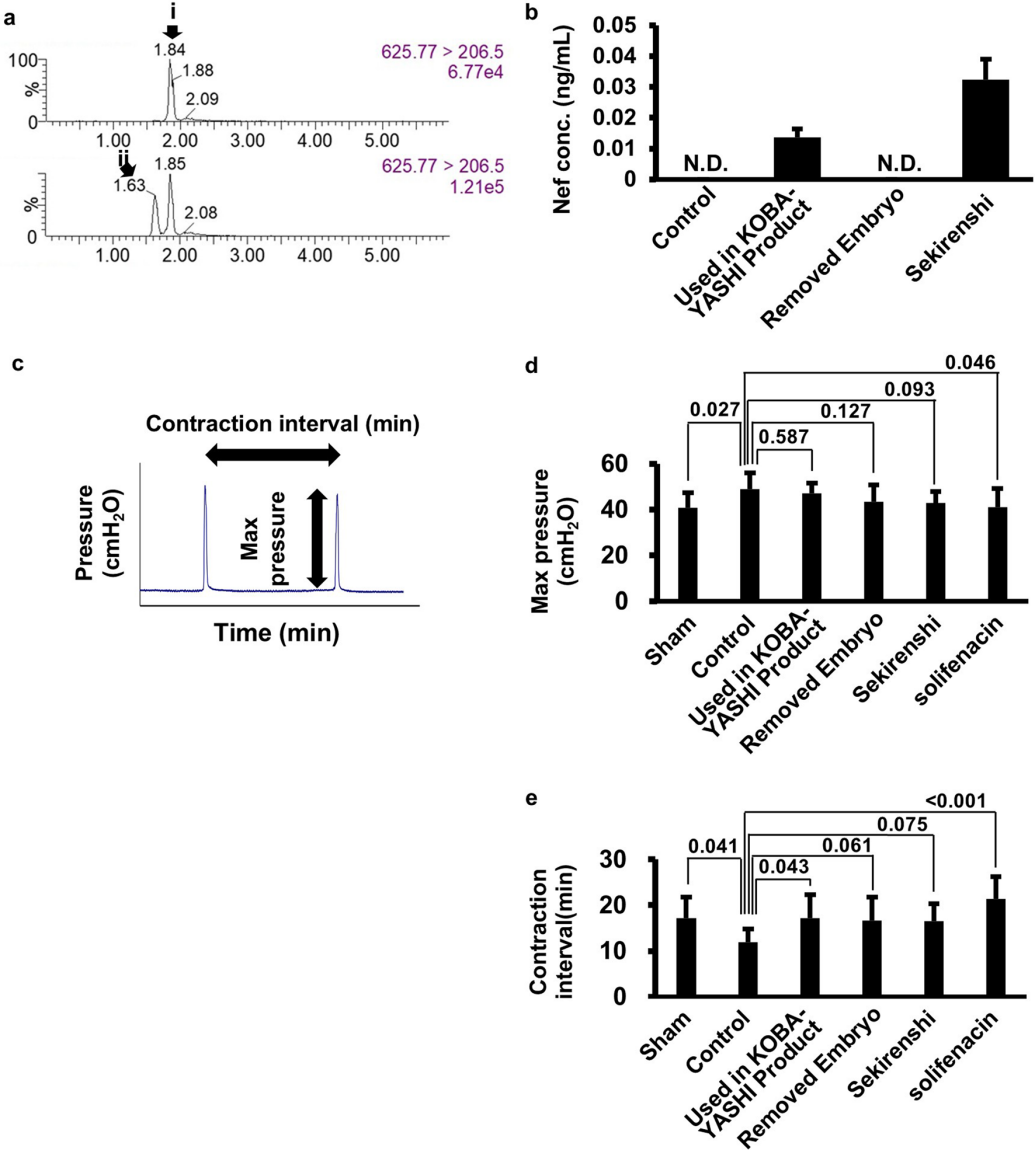
Effects of Nelumbo Seed extract on frequent-urination model rat. **a** LC/MS mass chromatogram of blank (upper) and Nelumbo Seed extract-administered rat (lower). MRM transition are 625.77 > 206.5. Peak i, dauricine (internal standard), ii, neferine. **b** Neferine concentrations in Nelumbo Seed extract-administered rat plasma. **c** Raw data of urinary pressure and interval measurement. **d**, **e** Maximum bladder contraction pressure (**d**) and bladder contraction interval (**e**) in frequent-urination model rats administered a different type of Nelumbo Seed extract. Values above the bars indicate *P* values obtained using the one-tailed Dunnett’s test in comparison with a control group, which was administered normal feed and subjected to pelvic congestion

The Nelumbo Seed materials used in this study were purchased by KOBAYASHI Pharmaceutical Co., Ltd. from a wholesaler. Because the amounts of materials were limited, the materials used for experiments, the results of which are presented in Figs. 1–3, were different. Information on Nelumbo Seed No.1 to 5 used in the rat excised bladder smooth muscle contraction inhibitory activity test and LC/MS chemical profiling is presented in Table 1. Information on Nelumbo Seed No. 6 to 9 used for neferine (Nef) quantification is presented in Table 2. The Nelumbo Seed materials used in the experiments on frequent urination model rats were purchased separately. Nelumbo Seed mentioned in Tables 2, 3 and Figs. 2, 3 were from a production site in Hunan Province, China.

**Table 1:** Nelumbo Seed materials used for the assay of inhibitory activity on excised rat bladder smooth muscle contraction and LC/MS metabolic profiling

No	Specific No. assigned by KOBAYASHI Pharmaceutical Co	Production site (Province, China)
1	NH160707TZ	Hunan
2	ZP160622FJ	Fujian
3	ZY16070803DW	Jiangxi
4	ZY1607080	Jiangxi
5	ZY16070804	Hunan

**Table 2:** Nelumbo Seed materials used for neferine quantitation

No		Embryo	Endocarp	Note	Nef conc. (μg/g)
6	Whole	Retained	Retained	Sekirenshi	315
7	Notch	Mostly removed	Retained	Derived from No. 6	Trace
8	Notch	Mostly removed	Retained		N.D
9	Notch	Completely removed	Completely removed		N.D

**Table 3:** Neferine concentration in the plasma of rats administered the Nelumbo Seed extract

Administration group	Nef conc. (ng/mL)
Control (*n* = 8)	N.D
Used in KOBAYASHI Pharmaceutical’s Product (*n* = 12)	0.014 ± 0.003
Removed Embryo (*n* = 12)	N.D
Sekirenshi (*n* = 12)	0.032 ± 0.007

### Preparation of Nelumbo Seed extracts for ex vivo and in vivo assays and LC/MS analysis

The extracts were prepared by an extract manufacturing company commissioned by KOBAYASHI Pharmaceutical Co., Ltd. Nelumbo Seeds (dry weight, 150–300 g) were crushed in a mill until they were reduced to approximately half of their original size. Five times the amount of distilled water was added and extraction was carried out at 95 °C for 1 h. The extract was filtered through a wire mesh with 0.109 mm openings and concentrated under a reduced pressure of 20 kPa or less at 60 °C or lower temperature using an evaporator. The concentrated liquid was then freeze-dried and the extract was powdered.

### Reagents

LC/MS grade acetonitrile, distilled water, and formic acid used for LC/MS analysis were purchased from Fujifilm Wako Pure Chemical Industries (Osaka, Japan). HPLC grade methanol, distilled water, trifluoroacetate, and ammonium acetate, used for HPLC analysis, were obtained from Nacalai Tesque (Kyoto, Japan). Neferine, used for the activity test, was purchased from Sigma-Aldrich (now, Merck KGaA, Darmstadt, Germany).

### Evaluation of bladder smooth muscle contraction inhibitory activity using excised rat bladder tissue

This test was commissioned by KOBAYASHI Pharmaceutical Co., Ltd. to the Shiga Research Institute of Nissei Baylis Co., Ltd. The experiment was approved by the Animal Experiment Committee of the testing facility (Approval number: 1610-21).

The Nelumbo Seed extracts used in this test were prepared by dissolving 300 mg of powdered extracts in 3 mL of water for injection (Fuso Pharmaceutical Industries, Ltd., Osaka, Japan).

Eight-week-old male Slc:SD rats (SPF grade; Japan SLC Co., Ltd.) were used for the experiment. After a 6-day quarantine period from receipt at the facility, the rats were acclimatized and used for the experiment. The rats were euthanized using excessive isoflurane anesthesia, and their abdomen was opened to remove the bladder. The bladder was cut vertically into strips, 2–3 mm wide and 5–10 mm long, to prepare the bladder specimens, with four specimens prepared from each animal. An organ bath (volume 10 mL, temperature 37 ± 1 °C) was filled with Krebs–Henseleit solution with a mixture of 95% O_2_ and 5% CO_2_ and suspended with a load of approximately 1 g. After equilibrating the specimen for 60 min, 50 μL of 2 mmol/L carbamylcholine solution (solvent: water for injection Fuso Pharmaceutical Industries, Ltd.) was added to the organ bath to get a final concentration of 10 μmol/L and the contractile response was recorded. After maximum contraction was achieved, the specimen was washed with the Krebs–Henseleit solution and allowed to rest for 15 min. During the rest period, the Krebs–Henseleit solution was replaced twice at 5 min intervals. Thereafter, the contractile response was recorded using the same procedure as followed for the first addition of carbamylcholine, and the specimen was washed with the Krebs–Henseleit solution and rested for 15 min. These procedures were repeated, and the contractile response to carbamylcholine was recorded for a total of three times. After recording the contractile response to the third carbamylcholine addition, the specimen was washed with the Krebs–Henseleit solution and rested for approximately 15 min. After the pause, 1 mL of each sample extract was added to the organ bath to obtain a final concentration of 10 mg/mL and incubated for 10 min. Carbamylcholine was then added, and the contractile response was recorded. The contraction width (mm) was measured using a ruler from the baseline immediately before the addition of carbamylcholine to maximum contraction width after the addition. The activity of each sample extract was defined as the decrease in contraction width upon addition of carbamylcholine after treatment with the Nelumbo Seed extract compared to the contraction width upon addition of carbamylcholine and without treatment with the Nelumbo Seed extract. Five samples of each extract were tested, and the average activity and standard deviation were calculated.

### Chemical profiling of Nelumbo Seed extract using liquid chromatography/mass spectrometry

Nelumbo Seed extract powder was dissolved in acetonitrile, adjusted to 1 mg/mL, and then filtrated through a Millex-LG filter (disc diameter: 13 mm; pore size: 0.2 μm; Merck Life Science, Darmstadt, Germany).

LC/MS analysis was performed using an Acquity UPLC system (Waters Co., Milford, MA, USA) and a Quattro-Premier XE (Waters). LC separation was carried out at 40 °C using a TSKgel-Amide 80 column (5 μm particle size, 2.0 mm i.d. × 150 mm; Tosoh Co., Tokyo, Japan). The mobile phase was delivered at a flow rate of 0.250 mL/min. A gradient elution profile consisting of solvent A (0.1% formic acid in distilled water) and solvent B (acetonitrile) was employed. The initial composition of the binary solvent was 0% solvent A, which was increased to 95% over 20 min. The solvent A was maintained at 95% for 5 min. Ten microliter of the sample solution was injected into the column. The mass spectrometer was set to MS scan mode and operated with an electrospray ionization source in positive ion mode. The capillary and cone voltages were set to 4.5 kV and 35 V, respectively. The scanning was performed over an *m/z* range of 100–1,000. The scan and interval times were set at 0.95 and 0.05 s, respectively. The MassLynx software Ver 4.1 (Waters) was used for the operation of the LC–MS system and data acquisition.

### Data analysis of LC/MS profile

The raw LC/MS data were converted to the netCDF format and uploaded to the MZmine 2.26 software [23]. The following operations were carried out using this software: i) scan by scan filtering (method: Savitzky–Golay filter; number of datapoints: 11), ii) mass detection (method: recursive threshold mass detector; noise level: 1.0 E^5^; minimum *m/z* peak width: 0.8; maximum *m/z* peak width: 1.5), iii) chromatogram builder (minimum time span: 0.05 min; minimum height: 3.0 E^5^; *m/z* tolerance: 0.5 or 500 ppm), iv) chromatogram deconvolution (method: local minimum search; chromatographic threshold: 1.0%; search minimum in RT range: 0.05 min; minimum relative height: 1.0%; minimum absolute height: 5.0 E^5^; minimum ratio of peak top/edge: 1; peak duration range: 0.0–15.0 min), v) isotopic peak grouper (*m/z* tolerance: 0.5 m*/z* or 500 ppm; retention time tolerance: 0.05 absolute min; monotonic shape: no; maximum charge: 2; representative isotope: lowest *m/z*), vi) alignment (method: join aligner; *m/z* tolerance: 0.5 m*/z* or 500 ppm; weight for *m/z*: 1; retention time tolerance: 0.05 min absolute; weight for RT: 1; isotope *m/z* tolerance: 0.5 m*/z* or 500 ppm; minimum absolute intensity: 1.0E^4^; minimum score: 70%), vii) export to csv format. This file contained information on 1,474 peaks. The exported data were opened in Microsoft Excel, and peaks that were detected in only one sample were deleted, leaving a total of 98 peaks. The excised rat bladder smooth muscle contraction inhibitory activity (%) was added to these data as an objective variable, and the peak IDs were modified for easy identification. These data were imported into SIMCA 13 (Sartorius Stedim Biotech, Goettingen, Germany), and orthogonal-projection to latent structure by means of partial least square (OPLS) was performed. The unit variance (UV) was used for scaling.

### Quantification of neferine in Nelumbo Seed materials

Nelumbo Seed No. 5–8 were ground using a Wonder Blender (Osaka Chemical Co., Ltd., Osaka, Japan). The powdered sample (200 mg) was weighted and 3 mL of 1.0 mol/L ammonium acetate (pH 10.0)/methanol (1/99, v/v) was added. After shaking for 15 min, the mixture was centrifuged at 5000 × *g* for 5 min and the supernatant was collected. This procedure was repeated twice, and the resulting extracts were mixed and made up to 10 mL with the extraction solvent. This sample was filtered through a Millex-LG filer (disc diameter: 4 mm; pore size: 0.2 μm; Merck Life Science) and subjected to HPLC.

The HPLC pump, column oven, autosampler, and fluorescence detector were L-2100, L-2300, L-2200, and L-2485, respectively (Hitachi High-Tech Corporation, Tokyo, Japan). The column used was an InertSustain Phenyl column (5 μm particle size, 4.6 mm i.d. × 250 mm length; GL Sciences Inc., Tokyo, Japan). The column oven temperature was set at 40 °C. The eluents used were 0.1% trifluoroacetic acid in distilled water as solvent A, and methanol as solvent B and autosampler solvent. The flow rate was 1.0 mL/min, and the proportion of solvent B was 40% from 0 to 18 min. The sample volume was 10 μL. The excitation and emission wavelengths for fluorescence detection were set to 284 and 326 nm, respectively, based on the results for a Nef standard solution analyzed using a HITACHI F-2500 fluorescence spectrophotometer (Hitachi High-Tech). The data were acquired using a D-2000 Elite (Hitachi, High-Tech).

### Effects of neferine in the Nelumbo Seed extract on frequent-urination model rat

The experiment was conducted at Southern Knights’ Laboratory, Co., Ltd. The experimental procedures were approved by the Institutional Animal Care and Use Committee of the University of the Ryukyus (Approval number: A2017012, A2017189). A rat model of frequent urination was prepared as follows [24]: SD rats were subjected to laparotomy under isoflurane inhalation anesthesia, and both common iliac veins and both uterine veins were ligated, and 50 mg of cefamezin was administered subcutaneously. In the sham group, both common iliac veins were not ligated, but were only dissected from the common iliac artery. After suturing, the rats other than those in the sham group were divided into the following five groups that were fed different feeds: i) ‘Control’: normal feed, ii) ‘Used in KOBAYASHI Pharmaceutical’s Product’: feed contained 0.25% extract of Nelumbo Seed, which is used in KOBAYASHI Pharmaceutical’s SRI formulation, with embryo not completely removed, iii) ‘Removed Embryo’: feed contained 0.25% extract of Nelumbo Seed, with embryo removed completely, iv) ‘Sekirenshi’: feed contained 0.25% extract of ‘Sekirenshi’, Nelumbo Seed in which the endocarp and embryo were not removed at all, v) ‘solifenacin’: feed contained 0.01% solifenacin. The rats were raised for two weeks. Thereafter, a catheter was inserted into the bladder through the urethra, while the rats were under light urethane anesthesia (0.6 g/kg, subcutaneous injection) and restrained. One hour after the anesthetic effect appeared, continuous cystometric measurements were performed while saline was injected through the indwelling bladder catheter at a rate of 3 mL/h. The maximum bladder contraction pressure and bladder contraction interval were used as evaluation indices [24, 25].

### Quantification of neferine in the plasma of model rats

To 490 μL of rat plasma, 10 μL of 500 ng/mL dauricine (PhytoLab GmbH & Co., KG, Vestenbergsgreuth, Germany) in methanol solution was added, followed by 500 μL of ethyl acetate. After shaking for 10 min, the mixture was centrifuged at 15,000 × *g* for 5 min and the supernatant was collected. Ethyl acetate (500 μL) was added to the remaining plasma layer and the mixture was shaken and centrifuged as described above. The collected supernatant was dried under a nitrogen stream. After removing the solvent, the residue was dissolved in 100 μL of acetonitrile/methanol (90/10, v/v) and filtered through a Millex-LG filter (4 mm, pore size 0.2 μm; Merck Millipore). LC/MS analysis was performed using an Acquity UPLC and a Quattro-Premier XE. LC separation was carried out at 40 °C using an Acquity UPLC BEH Amide column (1.7 μm particle size, 2.1 mm i.d. × 50 mm length; Waters). The mobile phase was delivered at a flow rate of 0.5 mL/min using a gradient elution profile comprising solvent A (0.1% formic acid, 10 mmol/L ammonium formate in methanol) and solvent B (0.1% formic acid, 10 mmol/L ammonium formate in acetonitrile/methanol (90/10, v/v). The composition of solvent B was 100% (0–0.5 min), 100–90% (0.5–1.5 min), 90–10% (1.5–2.0 min), 10% (2.0– 3.0 min), 10–100% (3.0–3.1 min), and 100% (3.1–6.0 min). The sample volume injected into the column was 10 μL. The mass spectrometer was set to the multiple reaction monitoring (MRM) mode and operated with an electrospray ionization source in the positive ion mode. The capillary voltage was set at 4.5 kV. MRM transitions (*m/z* of precursor ion > *m/z* of product ion) for Nef and dauricine measurement were 625.8 > 206.5 (for quantification), 625.8 > 121.3 (for confirmation), and 625.8 > 489.7 (for confirmation). The cone voltage was 56 V, and the collision energies were 34 (for 625.8 > 206.5), 68 (for 625.8 > 121.3), and 36 (for 625.8 > 489.7) eV, respectively. The dwell time for each MRM transition was 0.1 s. The MassLynx software Ver 4.1 (Waters) was used for the operation of LC–MS and data acquisition.

### Statistical analysis

The statistical significance of differences in the maximum bladder contraction pressure and bladder contraction interval between rats administered the Nelumbo Seed extract and control rats, which consumed normal feed and were subjected to pelvic congestion, was assessed with one-tailed Dunnett’s test performed using the R software.

## Results and discussion

### Bladder smooth muscle contraction inhibitory activity of Nelumbo Seed extract

First, we evaluated the inhibitory activity of Nelumbo Seed extract on bladder smooth muscle contraction using excised rat bladder tissue. This method measures the extent to which the contraction of the excised rat bladder smooth muscle upon treatment with carbamylcholine, a cholinergic agonist, is inhibited by pretreatment with the test samples. Figure 1a and Table 1 present an overview of the five types of Nelumbo Seed samples, their identification numbers and area of production. The inhibitory activities of the samples on excised rat bladder smooth muscle contraction are shown in Fig. 1b. Tolterodine, used as a positive control at a final concentration of 100 ng/mL, showed 68.7 ± 5.9% activity. The activity varied with the samples, with sample No. 2 showing the highest inhibition rate (27.8 ± 9.1%), followed by samples No. 3 and No. 4, which showed similar activities (15.5 ± 5.7 and 14.8 ± 2.6%, respectively). The inhibition rates of samples No. 1 and No. 5 were 6.5 ± 3.8 and 7.9 ± 4.8%, respectively. In addition, SRI extract prepared at the same extract powder weight concentration showed 68.0 ± 9.2% inhibition, which was higher than that of Nelumbo Seeds. These results indicate that the crude drugs in SRI other than Nelumbo Seed contain active ingredients that inhibit the contraction of bladder smooth muscle.

### Liquid chromatography/mass spectrometry profiles of Nelumbo Seed extract

Next, we determined the chemical profiles of the Nelumbo Seed extract using LC/MS analysis. With a reversed-phase octadecylsilyl (ODS) column, which is commonly used to measure the components of crude drug extracts, many peaks were obtained, without the components being retained in the column, resulting in insufficient peak separation (data not shown). Therefore, we performed hydrophilic interaction chromatography to increase the retention of hydrophilic substances that are difficult to retain with reversed-phase columns. An amide column was used, and the eluent gradient was set such that the acetonitrile content decreased from 100 to 0% over the run. The extract was dissolved in acetonitrile under the same initial conditions as the eluent at a concentration of 1 mg/mL, filtered through a membrane filter, and subjected to LC/MS. The resulting LC/MS total ion current chromatogram (TIC) revealed many peaks between retention times of 2–15 min. The peak at a retention time of 3.6 min was significantly higher for sample No. 2 and 4 than for sample No. 3 and 5. However, no significant differences in TIC were observed among samples. These results indicated that Nelumbo Seed samples used in this study were derived from the same original plant source of the crude drug, and exhibited similar component profiles. However, considering the differences in excised rat bladder smooth muscle contraction inhibitory activities among the samples, we hypothesized that these LC/MS profiles reflect information on the ingredients that affect the activities. Therefore, information on the retention time, *m/z*, and area of the detected peaks was extracted and subjected to multivariate analysis.

First, the raw LC/MS data were converted to the universal format ‘netCDF’, loaded into the free software, MZmine [23] (we used version 2.26.), and peak detection, deconvolution, and alignment were performed. Ninety-eight peaks were detected upon analysis of the LC/MS profile (Supplemental Table 1). OPLS [26], which is a multivariate regression analysis, was performed using the area of these peaks detected by LC/MS as explanatory variables and excised rat bladder smooth muscle contraction inhibitory activity of each sample as the objective variable. The explanatory variables were subjected to unit variance processing. As a result of the OPLS, a model with one latent variable and one orthogonal variable was constructed. The value of *R*^*2*^, which indicates the linearity of the regression equation, was 0.98, and that of *Q*^*2*^, which indicates the degree of model agreement during cross-validation, was 0.70, indicating the successful construction of a good regression model [26]. The Y-fit revealed a strong correlation between the predicted values of the excised rat bladder smooth muscle contraction inhibitory activity of each Nelumbo Seed extract calculated using the OPLS model and the experimentally observed values (Fig. 1d). Because the number of samples was very small, the OPLS model cannot be considered an accurate prediction model. However, we surmised that ingredients that are highly correlated with activity could possibly be extracted and, therefore, continued with subsequent analysis.

The variable importance in the projection (VIP) values, which represent the contribution of each LC/MS peak to the OPLS model construction [27], are shown in Fig. 1e. If the VIP value exceeds 1 and the confidence interval does not include 0 (within the error bar), the peak contributes to the OPLS model construction. Several peaks shown in Fig. 1e satisfied these conditions. The peak with the highest VIP value had a retention time of 10.1 min and an *m/z* of 625. Because the LC/MS analysis was conducted in the positive ion mode, the peak at *m/z* 625 corresponded to the protonated ion of a compound with a molecular weight of 624, and this molecular weight matched that that of Nef. Analysis of the commercially available Nef under the same LC/MS conditions revealed a matching retention time and *m/z* of the peak, confirming the identified component was Nef.

The mass chromatograms of *m/z* 625 for each Nelumbo Seed sample and SRI extract are shown in Fig. 1f. The areas of the peak at 10.1 min in these chromatograms are shown in Fig. 1g. The Pearson correlation coefficient between the peak areas (Fig. 1g) and the excised rat bladder smooth muscle contraction inhibitory activity of the Nelumbo Seed extract samples (Fig. 1b) indicated an extremely high correlation of 0.9887 (Fig. 1h).

Nef, a component of Nelumbo Seed, exhibits smooth muscle relaxant effects [28, 29]. However, most of these effects have been reported for vascular smooth muscles. Its contraction-inhibitory activity on bladder smooth muscle has not yet been confirmed. Therefore, we prepared an aqueous solution of Nef and examined its inhibitory activity on smooth muscle contraction in excised rat bladders and found that it was dose-dependent over a concentrations range of 1–25 μg/mL (Fig. 1i).

The VIP values suggested that the components corresponding to the ‘7.45 min, *m/z* 314’, ‘7.56 min, *m/z* 300’, and ‘7.99 min, *m/z* 286’ peaks might also have contributed to the inhibitory activity. These components are presumed to be intermediates, for example, *N*-methylnorcoclaurine, *N*-methylcoclaurine, and armepavine, in the Nef biosynthetic pathway, which might also show the activity. However, because the peak with the highest correlation was identified to be that for Nef and we could only procure Nef commercially, we focused on this compound in subsequent experiments.

### Variation factor for the neferine content in Nelumbo Seed materials

Next, we examined the factor causing variations in the Nef content of Nelumbo Seed materials. A photograph of Nelumbo Seed is presented in Fig. 2a. A slice of the seed is shown on the left and the embryo is indicated with an arrow. A slice of the seed with the pericarp removed is shown in the middle, and an untreated seed is shown on the right. A list of Nelumbo Seed materials used in this study to examine the causes of variation in the Nef content is presented in Table 2 and the morphological appearance of the seeds is shown in Fig. 2b. ‘Sekirenshi’ (No. 6) was the material in which the endocarp and embryo had not been removed at all. In No. 7 and 8, most of the embryo had been removed, and a considerable amount of endocarp was present whereas in No. 9, the endocarp and embryo were almost completely removed. We prepared extracts of these materials and quantified their Nef content using HPLC-FL (Fig. 2c). Nef was not detected in No. 8 and 9, and it was only present in trace amounts in No. 7. In contrast, the Nef content of No. 6 was 314 mg/g of Nelumbo Seed material (Table 2). These results indicated that the presence or absence of embryos is a factor in the variation of the Nef content in Nelumbo Seed. The presence of Nef in Nelumbo Seed embryos was demonstrated by Furukawa [30] and has been widely reported [31]. Although the materials used in the experiment, the results of which are presented in Fig. 1, contained embryos, the exact contents of embryos were unknown. We show that the Nef content of Nelumbo Seed materials varies considerably depending on the processing conditions.

### Effects of neferine in Nelumbo Seed extract on frequent urination model rat

Next, we evaluated the efficacy of Nelumbo Seed and Nef using a rat model of frequent urination. We created a pelvic congestion frequent urinary model by ligating the common iliac and uterine veins of the rats [24]. The rats were fed a normal diet or a diet supplemented with Nelumbo Seed extract (0.25% by weight) for 13 days. The positive control group was fed a normal diet supplemented with 0.01% solifenacin. To confirm that there was a difference in the plasma levels of Nef among the rats administered the three types of Nelumbo Seed extract, the Used in KOBAYASHI Pharmaceutical’s Product, Removed Embryo, and Sekirenshi, blood was collected after sacrifice and the plasma was subjected to LC/MS (Fig. 3a). Nef was not detected in the control rats fed normal feed or the Removed Embryo. Nef concentrations of 0.014 and 0.032 ng/mL were detected in rats administered the Used in KOBAYASHI Pharmaceutical’s Product and Sekirenshi, respectively. (Fig. 3b and Table 3). Therefore, it was confirmed that the plasma concentration of Nef increased to detectable levels in rats administered the Used in KOBAYASHI Pharmaceutical’s Product and Sekirenshi. The concentration of Nef was too low to be detected in rats administered SRI (data not shown).

We measured the parameters described below before sacrificing the rats for Nef quantification. The rats were lightly anaesthetized with urethane and restrained, and a catheter was inserted into the bladder via the urethra. Intravesical pressure was continuously measured while injecting saline (Fig. 3c), and the maximum bladder contraction pressure and bladder contraction interval were calculated (Fig. 3d, e). Compared with the control group, which consumed normal feed and was subjected to pelvic congestion, the maximum bladder contraction pressure significantly decreased in the sham group, which was not subjected to pelvic congestion (*P* = 0.027), and in the positive control group, which was administered solifenacin (*P* = 0.046). In contrast, the group administered the Nelumbo Seed extract showed a decrease in contraction pressure compared to the control group. In particular, the group administered the Sekirenshi extract showed the largest decrease among the groups administered the Nelumbo Seed extract (*P* = 0.587, vs. Used in KOBAYASHI Pharmaceutical’s Product; *P* = 0.127, vs. Removed Embryo extract; *P* = 0.093, vs. Sekirenshi extract), indicating that the Sekirenshi extract may be a suitable component for SRI. However, these decreases were not statistically significant, and the group administered the Removed Embryo, for which Nef was not detected in the plasma, showed a tendency for the pressure to decrease more than that in the group administered the Used in KOBAYASHI Pharmaceutical’s Product, in which Nef was detected.

The bladder contraction intervals were longer in the sham group (*P* = 0.041) and positive control group, which was administered solifenacin (*P* < 0.001), indicating the successful establishment of the frequent urination model [25]. The groups administered the Nelumbo Seed extract also tended to recover to the same level as the sham group (*P* = 0.043, Control vs. Used in KOBAYASHI Pharmaceutical’s Product; *P* = 0.061, vs. Removed Embryo extract; *P* = 0.075, vs. Sekirenshi extract). However, no clear difference was observed among the groups administered the Used in KOBAYASHI Pharmaceutical’s Product, Removed Embryo, and Sekirenshi extracts.

These results do not support the effectiveness of Nef against frequent urination because the Nef content of the Nelumbo Seed extract and blood concentration of Nef in the rats did not affect the bladder contraction intervals in the frequent urination model rats. However, the frequent urination model rats administered the Sekirenshi extract showed the largest decrease among the rats administered the Nelumbo Seed extract, indicating that Nef in Nelumbo Seed might contribute to the improvement of urination disorder. Moreover, the effectiveness of Nelumbo Seed has not been ruled out, and other active ingredients might be present in it. We believe that it would be worthwhile to verify other indicators and frequent urination models.

In this study, we observed that Nef is an active ingredient in Nelumbo Seed that inhibits bladder smooth muscle contraction. We quantified the Nef content in Nelumbo Seed materials processed using different methods and found it to be high in Sekirenshi, in which the embryo and pericarp were not removed at all. The concentration of Nef in rat plasma increased upon administering the Sekirenshi extract but it was not detected when Removed Embryo was administered. The maximum bladder contraction pressure tended to decrease in Sekirenshi extract-treated rats compared with that in the group not treated with the extract. Our results indicate that although Nef in Nelumbo Seed does not clearly improve frequent urination, it might contribute to the improvement of urination disorder.

## Supplementary Information

Below is the link to the electronic supplementary material.

Supplementary file1 (XLSX 20 KB)
